# Neuropathic pain following spinal cord hemisection induced by the reorganization in primary somatosensory cortex and regulated by neuronal activity of lateral parabrachial nucleus

**DOI:** 10.1111/cns.14258

**Published:** 2023-05-11

**Authors:** Jing Li, Chao Tian, Shiyang Yuan, Zhenyu Yin, Liangpeng Wei, Feng Chen, Xi Dong, Aili Liu, Zhenhuan Wang, Tongrui Wu, Chunxiao Tian, Lin Niu, Lei Wang, Pu Wang, Wanyu Xie, Fujiang Cao, Hui Shen

**Affiliations:** ^1^ Department of Orthopedics Tianjin Medical University General Hospital Tianjin China; ^2^ School of Biomedical Engineering Tianjin Medical University Tianjin China; ^3^ Department of Cellular Biology, School of Basic Science Tianjin Medical University Tianjin China; ^4^ Department of Physiology Zhuhai Campus of Zunyi Medical University Zhuhai China; ^5^ Innovation Research Institute of Traditional Chinese Medicine Shandong University of Traditional Chinese Medicine Jinan China

**Keywords:** in vivo calcium image, lateral parabrachial nucleus, neuropathic pain, primary somatosensory cortex, Spinal cord hemisection

## Abstract

**Aims:**

Neuropathic pain after spinal cord injury (SCI) remains a common and thorny problem, influencing the life quality severely. This study aimed to elucidate the reorganization of the primary sensory cortex (S1) and the regulatory mechanism of the lateral parabrachial nucleus (lPBN) in the presence of allodynia or hyperalgesia after left spinal cord hemisection injury (LHS).

**Methods:**

Through behavioral tests, we first identified mechanical allodynia and thermal hyperalgesia following LHS. We then applied two‐photon microscopy to observe calcium activity in S1 during mechanical or thermal stimulation and long‐term spontaneous calcium activity after LHS. By slice patch clamp recording, the electrophysiological characteristics of neurons in lPBN were explored. Finally, exploiting chemogenetic activation or inhibition of the neurons in lPBN, allodynia or hyperalgesia was regulated.

**Results:**

The calcium activity in left S1 was increased during mechanical stimulation of right hind limb and thermal stimulation of tail, whereas in right S1 it was increased only with thermal stimulation of tail. The spontaneous calcium activity in right S1 changed more dramatically than that in left S1 after LHS. The lPBN was also activated after LHS, and exploiting chemogenetic activation or inhibition of the neurons in lPBN could induce or alleviate allodynia and hyperalgesia in central neuropathic pain.

**Conclusion:**

The neuronal activity changes in S1 are closely related to limb pain, which has accurate anatomical correspondence. After LHS, the spontaneously increased functional connectivity of calcium transient in left S1 is likely causing the mechanical allodynia in right hind limb and increased neuronal activity in bilateral S1 may induce thermal hyperalgesia in tail. This state of allodynia and hyperalgesia can be regulated by lPBN.

## Graphical abstract

After spinal cord hemisection, contralateral hind limb and tail of mice are sensitive to injurious stimuli. In this state of pain hypersensitivity, significant differences happen in calcium transient between the left and right primary somatosensory cortex. Chemogenetic activation or inhibition of the neurons in the lateral parabrachial nucleus, pain hypersensitivity was increased or alleviated.

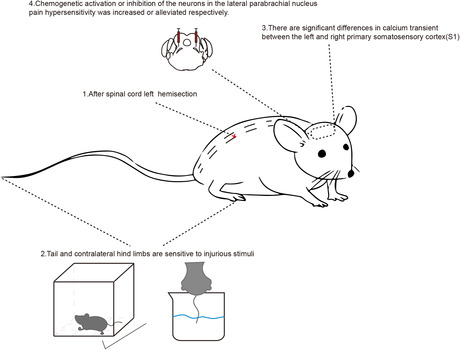

## 1 INTRODUCTION

Spinal cord injury causes varying degrees of sensory and motor dysfunction and is often accompanied by complex and diverse complications.^1^ Chronic neuropathic pain, as one of the common complications after SCI, makes huge and negative effect on quality of life, including sleep, mood, and participation in activities as well as employment.^2^, ^3^, ^4^, ^5^ It has been reported that nearly one‐third of people with SCI develop aberrant feeling, but available therapeutic strategies are limited up to now.^5^, ^6^, ^7^ Indeed, at‐level or below‐level neuropathic pain after spinal cord injury is believed likely to reflect different underlying mechanisms, which are most likely related to circuit plasticity that occur at various levels of the nervous system, involving structural changes in dorsal horn neurons^8^ and supraspinal mechanisms.^9^, ^10^ A great deal of research have provided strong evidence to support the notion that changes in the brain are associated with the presence of neuropathic SCI pain, such as alterations of neuronal firing and biochemical changes in the thalamus,^11^, ^12^ differential expression of receptors in amygdala, anterior cingulate cortex, and periaqueductal gray.^13^ In addition to these changes in subcortical structures, neuropathic pain following SCI has also been reported to be associated with increased excitability within cortical regions. Previous studies have uncovered a strong correlation between primary somatosensory cortex reorganization and aberrant feeling by investigating S1 reorganization in arm amputations with phantom sensation.^14^, ^15^ Functional magnetic resonance imaging (fMRI) indicates profound reorganizations of the intact sensory‐motor cortex after unilateral spinal cord injury.^16^ And increased activation of contralateral S1 is a key feature of behavioral neuropathic pain in spinally injured rats.^17^ Recently, two‐photon in vivo imaging and genetically encoded calcium sensor advances make it possible to explore single neuron activity and map brain network of great populations of neurons over weeks to months after spinal cord injury.^18^, ^19^, ^20^ A study has shown that peripheral nerve pain can induce an increase in synchronized neuronal activity and connectivity within contralateral S1, which indicates the formation of pain microcircuits.^21^ However, few relevant research has yet observed the relationship between central neuropathic pain with calcium activity in S1, such as allodynia and hyperalgesia after SCI.^22^ Allodynia (pain due to a stimulus that does not usually provoke pain) and hyperalgesia (increased pain from a stimulus that usually provokes pain) are prominent symptoms in neuropathic pain.^23^ Though mechanical allodynia/hyperalgesia and thermal allodynia/hyperalgesia have separate mechanisms, their maladaptive central changes may be the same.

Furthermore, the lateral parabrachial nucleus, as one of major targets of spinal projection neurons in the brain, play a significant role in development and maintenance of neuropathic pain‐like behavior.^24^, ^25^ Pain is a multidimensional experience with sensory‐discriminative, affective‐motivational, and cognitive‐evaluative components.^26^ And the lPBN is a pivotal nucleus in pain emotional and cognitive networks.^26^ It has been shown in rats that most of lamina I projection neurons in the spinal cord project to the PBN.^8^ And lPBN neurons play a key role in processing nociceptive information originating from the ascending spinoparabrachial pathway.^27^ Indeed, the majority of lPBN excitatory neurons respond to noxious stimuli and their efferents to midbrain and forebrain contribute to appropriate behavioral responses for survival.^28^, ^29^ However, under the condition of pathological stimulation after LHS, the role of lPBN in the presence of central neuropathic pain has not been studied.

By withdrawal threshold test and tail immersion test, we identified mechanical allodynia of right hind limb and thermal hyperalgesia of tail after left LHS. During these tests, we examined calcium activity in single neurons in bilateral S1 by in vivo two‐photon imaging in mice. Mechanical pain stimulation of the right hind limb increased contralateral S1 calcium activity while thermal pain stimulation of the tail increased bilateral S1 calcium activity, and this effect was slightly enhanced after LHS. Then we observed long‐term spontaneous calcium activity after left spinal cord hemisection injury. Although the amplitude of calcium transient was decreased, the functional connectivity in the left S1 excitatory neurons was gradually increased and calcium transient in right S1 was increased more dramatically, which including frequency, function connectivity, and synchronization.^30^ Furthermore, we investigated the electrophysiology of lateral parabrachial nucleus. We reported that excitatory neurons in lPBN were activated after LHS. Since the hemi‐spinal cord sends projections to the bilateral lPBN,^27^, ^31^ we bilaterally activated or inhibited the lPBN to reveal the requirement of it in the maintenance neuropathic pain after LHS. The activation of lPBN excitatory neurons in wild mice could induce mechanical allodynia of right hind limb and thermal hyperalgesia of tail whereas inhibition in injured mice could alleviate this negative state. Our results demonstrate that allodynia of right hind limb may be associated with enhanced left S1 function connectivity while hyperalgesia of tail may be induced by bilateral S1 abnormal neuronal activity and this state can be simulated or mitigated by modulating lPBN excitatory neurons.

## 2 MATERIALS AND METHODS

### 2.1 Animal

Adult male C57BL/6 mice (8–10 weeks, 20–25 g, Institute of Zoology in Chinese Academy of Sciences) were used in the present study. The total number of animals is about 80 and sample quantity for different groups were described in detail in manuscript. All animals were randomly divided into experimental (LHS) and control groups. All mice were given free access to water and food under standard laboratory conditions (suitable temperature and humidity room with twelve‐hour day and night cycles). Simplifying the process to minimize the suffering of animals in the experiment and reduce the number of animals on the premise of obtaining sufficient experimental data. All experimental protocols and procedures were approved by the Animal Care and Use Guidance of Tianjin Medical University.

### 2.2 Viruses

The following viruses were used for experimentation: rAAV‐CaMKIIa‐GCaMp6f‐WPRE‐hGH, pAAV‐CaMKIIa‐hM3D (Gq)‐mCherry‐WPRE, pAAV‐CaMKIIa‐mCherry‐WPRE pAAV‐CaMKIIa‐hM4D (Gi)‐mCherry‐WPRE, Viruses were purchased from OBIO TECHNOLOGY (SHANGHAI) CORP., LTD.

### 2.3 Spinal cord injury and post‐operative care

The left spinal cord hemisection models were used in the study. Mice were anesthetized with an isoflurane‐oxygen mixture (1.5% vol isoflurane/vol O_2_) and ophthalmic ointment was applied to the eyes. The thoracic spinal skin was exposed and shaved with a razor. Iodine was used for skin sterilization. A longitudinal incision around 2 cm was made over T9–T10 level of the vertebrae. The fascia and muscle over T9–T10 were removed with a curved scissors. Mice were immobilized on a stereotaxic apparatus and laminectomy was performed at T10 segment. Then the spinal cord was exposed and the left (ipsilateral) was completely hemisected by a microscalpel under stereomicroscopy.^32^ The sham group received laminectomy only. The overlaying muscle layers were sutured and the skin was stapled closed. Body temperature was maintained at 37 ± 0.5°C with an electric heated pad until mice were fully awake. To prevent infection and inflammation after surgery, cyclosporine (15 mg/kg, subcutaneous) was injected once daily for consecutive 3 days.^33^ The bladder of the mice was manually voided twice daily until normal voiding reflexes returned. The surgery was performed by one person for homogeneous injury.

### 2.4 CatWalk gait analysis

Locomotor function was evaluated with the CatWalk gait analysis assay.^34^ Mice were trained daily during 2 weeks before the surgery to ensure the quality of the data obtained from the CatWalk analysis. Up to 5 runs were recorded per mouse, and analysis was performed on 3 of these runs showing an uninterrupted crossing of the glass runway within 1 to 2 s. Data on gait were acquired and analyzed with the CatWalk 7.1 software program. Recovery of weight support was required to be able to perform gait analysis. All animals recovered weight support by the first week after surgery. Gait parameters showing an SCI‐induced deficit specific to the ipsilateral side (side of injury) were selected for analysis: paw print area (surface of the paw in contact with the glass runway) and stance duration (duration of paw contact with glass runway). Different limbs contact with glass runway were represented by different colors. All of these parameters were measured as an average for the step cycles.

### 2.5 Behavioral test

Withdrawal threshold test: Using Semmes‐Weinstein monofilaments (North Coast Medical, Inc.), the nociceptive threshold of the intact hind paw to mechanical stimulus was examined.^35^ Animals were placed in a box with a wire grid floor and habituated to the environment for 10 to 15 min. Filaments were applied in either ascending or descending strengths (Dixon's up‐down method), and each filament was applied × times/only once for a maximum of 2 s. Paw withdrawal during the stimulation was considered a positive response, and the 50% response withdrawal threshold was calculated as the strength of filament that was given.

Tail immersion test: Mice were habituated to mice restraints 15 min for 5 days before testing. Tails were immersed 3 cm into a water bath at 46°C, 48°C or 52°C, and the latency of tail flick was measured three times per temperature with a one‐min interval between trials.

### 2.6 Stereotaxic injections and implantation of custom‐made head clamps

For in vivo two‐photon imaging:mice were anesthetized with an isoflurane‐oxygen mixture (1.5% vol isoflurane/vol O2) and immobilized on a stereotaxic frame and body temperature was maintained at 37 ± 0.5°C with an electric heated pad during surgery. A 1 cm incision was made on scalp with an eye scissor. A craniotomy (5 mm × 5 mm) was made above the left (ipsilateral) or right (contralateral) somatosensory cortex (AP: −0.8 to −1.3 mm; ML: ±1 to 1.5 mm) with a dental drill and dura was removed with an eye forceps. Virus injection was performed using a glass micropipette. For calcium imaging, A volume of ~200 nL of rAAV‐CaMKIIa‐GCaMp6f‐WPRE‐hGH was slowly injected into layer 2/3 neuron of somatosensory cortex (AP: −1.0 mm; ML: ±1.5 mm; DV: 0.25–0.35 mm). After injection, the micropipette was kept in cortex for at least 5 min to prevent backflow and then pulled out very slowly. The coordinates were referred to *the George Paxios and keith B. J. Franklin Mouse Brain Atlas, second edition*. Then the cranial window was covered with a 5 mm‐diameter cover glass, and then a headplate was glued to the skull using dental cement for mice immobilized during calcium imaging. Mice were put back to their home cages and wait for 3–4 weeks before calcium recording. After surgery, carprofen (5 ug/g bodyweight, i.p.) was used for 3 days.^36^

For viral labeling: pAAV‐CaMKIIa‐mCherry‐WPRE was bilaterally injected into the lPBN in WT mice (AP, 5.05 mm; ML, ± 1.28 mm; DV, 3.27 mm relative to bregma). After three weeks' expression, we performed left spinal cord hemisection in injured group and laminectomy in sham group. A week later, acute brain slice was prepared for patch clamp recording.

For pharmacogenetic stimulation: pAAV‐CaMKIIa‐hM3D (Gq)‐mCherry‐WPRE or pAAV‐CaMKIIa‐mCherry‐WPRE was bilaterally injected into the lPBN in mice. After three weeks expression, Clozapine Noxide (CNO, dissolved in saline to a concentration of 0.5 mg/mL. Enzo Life Sciences, Inc.) was injected intraperitoneally. 1 h later, we performed behavioral measurements and immunofluorescence.

For pharmacogenetic suppression: pAAV‐CaMKIIa‐hM4D (Gi)‐mCherry‐WPRE or pAAV‐CaMKIIa‐mCherry‐WPRE was bilaterally injected into the lPBN in mice. After three weeks' expression, we performed left spinal cord hemisection in injured group and laminectomy in sham group. A week later, behavioral tests were performed before and 1 h after the CNO injection. Immunofluorescence assay was also performed after CNO injection.

### 2.7 Immunofluorescent staining

Staining with CaMKIIa was used to assess the expression of Gcamp6f in excitatory neurons within S1 and staining with c‐Fos was used to assess the effect of operations or pharmacogenetic manipulations on neuronal activity in the lPBN. For c‐Fos quantification, mice were performed operations or CNO intraperitoneal injection. The former was perfused 7 days after surgery and the latter was perfused 2 h after injection. Mice were deeply anesthetized with isoflurane and then transcardially perfused with 0.1 M PBS and 4% paraformaldehyde in PBS. The brain was then removed and fixed in 4% paraformaldehyde buffer at 4°C overnight. After fixation, the brain was performed gradient dehydration in 20% sucrose and 30% sucrose (wt/vol) at 4°C. Frozen sections (25–35 μm) were cut on a cryostat (Leica CM 1950), and mounted on slides immediately after sectioning. After permeabilization and blocking for 1 h in 5% bovine serum albumin (BSA) and 0.5% Triton X‐100 in PBS, the slices were incubated at 4°C overnight with primary antibody diluted in BSA. (anti‐c‐Fos, 1:500, rabbit, Cell Signaling Technology; anti‐CaMKIIα, 1:500, rabbit, Abcam). After PBS wash, slices were subsequently incubated with secondary antibody in diluted BSA at room temperature for 3 h. (anti‐Rabbit Alexa Fluor 488, 1:1000, Invitrogen; anti‐Rabbit Alexa Fluor 647, 1:1000, Invitrogen). Next, nuclei were stained with DAPI, and confocal images were captured on a Zeiss LSM 800 microscope with a 10× or 20× objective (N.A. 1.0; Carl Zeiss). Cell counting was carried out manually using Image J‐win64.

### 2.8 Two‐photon imaging

Images of spontaneous calcium activity were obtained with a two‐photon microscopy, calcium activity of neurons was imaged using a Ti: Sapphire laser (model “Mai‐Tai Deep See”, Spectra‐Physics) and a water‐immersion objective (40×, numerical aperture = 1.10, Nikon), the excitation wave length was 910 nm. Image sessions were acquired at 512 × 512 pixels arrays and images were streamed at 30 Hz. For each imaging session, mice head were stabilized on a custom‐made clamp. Calcium imaging was conducted at pre, 1d, 3d, 7d, 14d, 28d after spinal cord injury.

### 2.9 Image processing and quantification

Image analysis of calcium imaging was performed using ImageJ software and custom‐written scripts in MATLAB (version 2014a; MathWorks; MA). Movies focal plane displacements were corrected using Mosaic Package in MATLAB. For the analysis of neuron calcium signal, we delineated cell edges carefully. We manually outlined 25 cells per field of vision and completely silent neurons (and/or those not expressing GCaMP6f) would be avoided using our approach. To quantify calcium signal, somatic regions of interest (ROIs) fluorescence intensity were determined with a semi‐automated algorithm that correlated the fluorescence intensity between adjacent pixels. The calcium signals of each ROI were averaged, and ROIs were confirmed again by visual inspection. All pixels within the cell‐based ROIs were averaged as relative percentage changes (∆F/F) after background subtraction for each neuron, calcium transients were represented as changed in fluorescence intensity. The synchronization and functional connectivity were described previously.^37^

### 2.10 LPBN brain slice preparation

Adult male mice were anesthetized with isopentane (4% in O_2_) and quickly decapitated in accordance with national and medical institute guidelines. To increase the viability of the cells in the brain slices, the brain tissue was placed in ice‐cold artificial cerebral spinal fluid (ACSF) containing (in mM): 120 NaCl, 2.5 KCl, 1.25 NaH_2_PO_4_, 26.2 NaHCO_3_, 1.3 MgSO_4_, 11 glucose, and 2.5 CaCl_2_ (equilibrated with 95% O_2_ + 5% CO_2_). Brain slices containing lPBN region with a thickness of 300 μm were sectioned at a coronal angle. The slices were transferred to ACSF at 33°C for incubation for at least 30 min and then allowed to recover for at room temperature for 1 h until the experiment was conducted.

### 2.11 Patch clamp recording

Electrophysiological recordings were performed as previously described.^38^ The neurons were visualized using a microscope with infrared differential Interferometric contrast optics (IR‐DIC) and fluorescent optical components, and the neurons in the lPBN brain region were distinguished by their relative positions in the posterior brain of the mouse brain map and by being fluorescentially labeled. Patch electrodes were prepared with capillary glass using P97 micropipette puller. In voltage‐clamp whole‐cell recording mode, patch pipettes (3–5 MΩ) were filled with a solution containing (in mM) 140 Cs‐methanesulfonate, 1 MgCl_2_, 10 HEPES, 0.2 EGTA, 5 NaCl, 2 MgATP, 0.3 NaGTP, and 2 QX 314‐ bromide adjusted to pH 7.2 with CsOH, osmolarity adjusted to 275–280 mOsm. sEPSCs and sIPSCs were recorded at holding membrane potentials of −70 and 0 mV, respectively. In current‐clamp whole‐cell recording mode, patch pipettes (3–5 MΩ) were filled with a solution containing (in mM) 125 K‐gluconate, 10 KCl, 5 NaCl, 0.2 EGTA, 10 HEPES, 2 Mg‐ATP, 0.3 Na‐GTP adjusted to pH 7.2 with KOH, osmolarity adjusted to 275–280 mOsm. Intrinsic neuronal excitability was calculated by injection of depolarizing current (ramp，0.14pA/ms) after hyperpolarizing the membrane (−60 pA for 1 s). In cell‐attached recordings, spontaneous neuronal firing patterns were assessed in voltage‐clamp mode (V = 0 mV) and in current‐clamp mode (I = 0 pA). Digital signals were sampled using a MultiClamp 700 B amplifier, filtered at 1 kHz, and digitized at 10 kHz. pCLAMP 10 software was used for further analysis.

### 2.12 Statistical analysis

All data was subject to tests for normal distribution (Kolmogorov–Smirnov test). The statistical analysis was performed using GraphPad Prim 9 (GraphPad Software Inc., La Jolla, CA). Data were presented as Mean ± SEM and tested by unpaired *t*‐tests, paired *t*‐tests, nonparametric test, or analysis of variance (ANOVA). Due to the hemisection and craniotomy performed at the same animal for experiment, some mice died in the middle of experiment (no more than 5 percent).

## 3 RESULTS

### 3.1 Spinal cord hemisection causes mechanical allodynia and thermal hyperalgesia in mice

**FIGURE 1:**
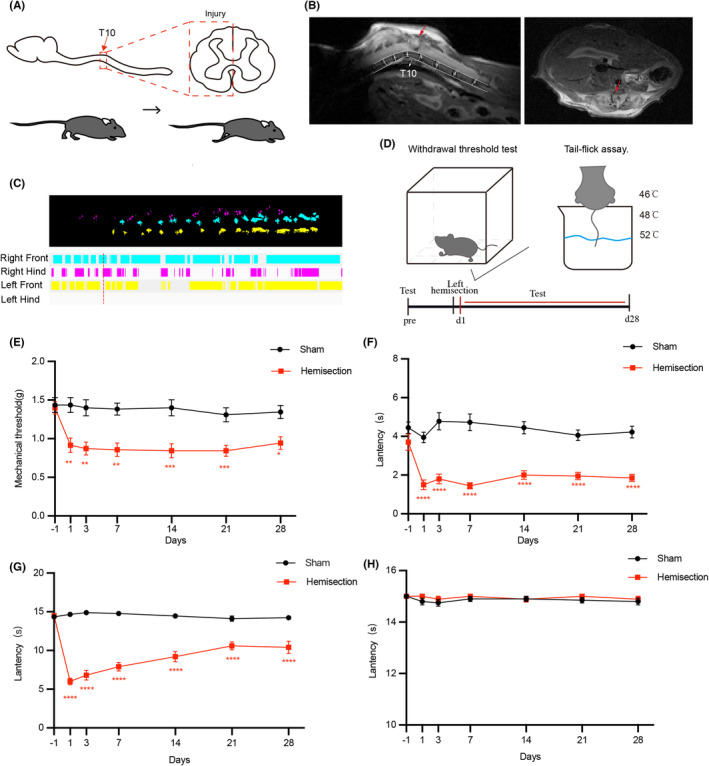
Chronic allodynia and hyperalgesia following left spinal cord hemisection in mice. (A) Schematic for left spinal cord hemisection at T10. (B) Left: the sagittal plane of MRI (the white arrow represented T10 vertebrae and the red arrow represents site of LHS). Right: the cross‐section of spinal cord of MRI (the red arrow represented site of LHS). (C) CatWalk Gait Analysis of paw print area and stance duration. (D) Protocol for the withdrawal threshold test and the tail‐flick assay. (E) Mechanical threshold was declined after LHS in injured group (Sham group *n* = 11 mice; Injured group *n* = 14 mice). (F, G) Latency to tail flick was significantly declined in water at 52 and 48°C after LHS (Sham group *n* = 9 mice; Injured group *n* = 10 mice). (H) There was no alteration in latency to tail flick in water at 46°C after LHS. (Sham group *n* = 9 mice; Injured group *n* = 10 mice). All data were presented as mean ± s.e.m. and error bars represented s.e.m. All the data were tested for normal distribution followed by two‐way ANOVA. *p < 0.05, **p < 0.01, ***p < 0.001 and ****p < 0.0001.

To evaluate the impact of LHS to the responses of mice to mechanical and temperature stimuli, we made left spinal cord hemisection at T10 on C57 mice under the microscope (Figure 1A). The sagittal plane of MRI showed the exact site of injury according to anatomic markers^39^ (the highest natural curvature of the spine) and the cross‐section of spinal cord showed left spinal cord injury but right intact (Figure 1B). The right hind paw print area and stance duration was normally detected by CatWalk Gait Analysis system, but left was disappeared, indicating that the injured group completely lost the motor function in left hind limb but their contralateral side was intact. (Figure 1C and Video S1). The sham group was only performed laminectomy while the spinal cord was intact, had sound functions. We performed mechanical and temperature stimulation‐related behavioral tests before and at different periods after LHS (Figure 1D). In Semmes‐Weinstein monofilaments test, we found that the withdrew threshold of the right hind paw from injured mice was declined whereas the sham group was normal (Figure 1E). Next, we immersed the tails of mice in water at 46, 48, and 52°C to test their responses to temperature stimuli. To prevent scalding, the maximum soaking time was no more than 15 s at a time. We found that in comparison with sham group, the tail flick latency (TFL) of injured mice was significantly declined in water at 48 and 52°C (Figure 1F,G), but there was no difference in water at 46°C (Figure 1H). The 52°C and 48°C water was harmful stimuli to mice, both of which could cause pain. These data suggest that after left spinal cord hemisection, the mechanical threshold of right hind limb is declined and tail reacts more violently to noxious thermal stimulation, which indicate mechanical allodynia in right hind limb and thermal hyperalgesia in tail. In some respects, this model is similar to peripheral nerve pain model, Such as the maintenance of neuropathic pain and the production of mirror image pain.^22^, ^27^, ^28^, ^40^

### 3.2 The S1 neuronal calcium activity are closely related to pain stimuli, which has anatomical correspondence

**FIGURE 2:**
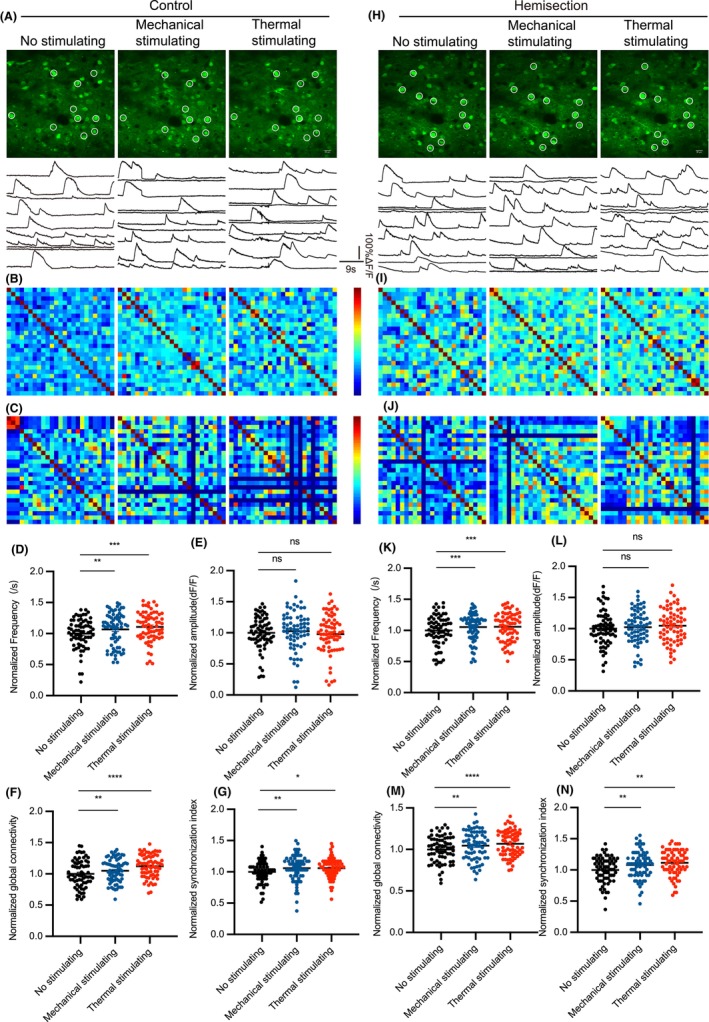
Mechanical pain stimulation of the right hind limb and thermal pain stimulation of the tail increased left S1 neuronal activity. (A, H) Example images of calcium transients as well as their neuronal calcium waves within left S1 in three phases before and after LHS. Circles represent typical neurons in individual microscope FOV. (B, I) Representative correlation matrix quantifying functional connectivity between each neuron and every other neuron within left S1 before and after LHS. (C, J) Representative correlation matrix quantifying network synchronization between each neuron and every other neuron within left S1 before and after LHS. (D–G) Frequency, amplitude, functional connectivity, and network synchronization of left S1 neuronal calcium transient waves in three phases before LHS (*n* = 1725 neurons per 7 mice). (K–N) Frequency, amplitude, functional connectivity, and network synchronization of left S1 neuronal calcium transient waves in three phases after LHS (*n* = 1725 neurons per 7 mice). All data were presented as mean ± s.e.m. and error bars represented s.e.m. All the data were tested for normal distribution followed by paired *t*‐test or nonparametric tests (Wilcoxon test). *p < 0.05, **p < 0.01, ***p < 0.001 and ****p < 0.0001.

**FIGURE 3:**
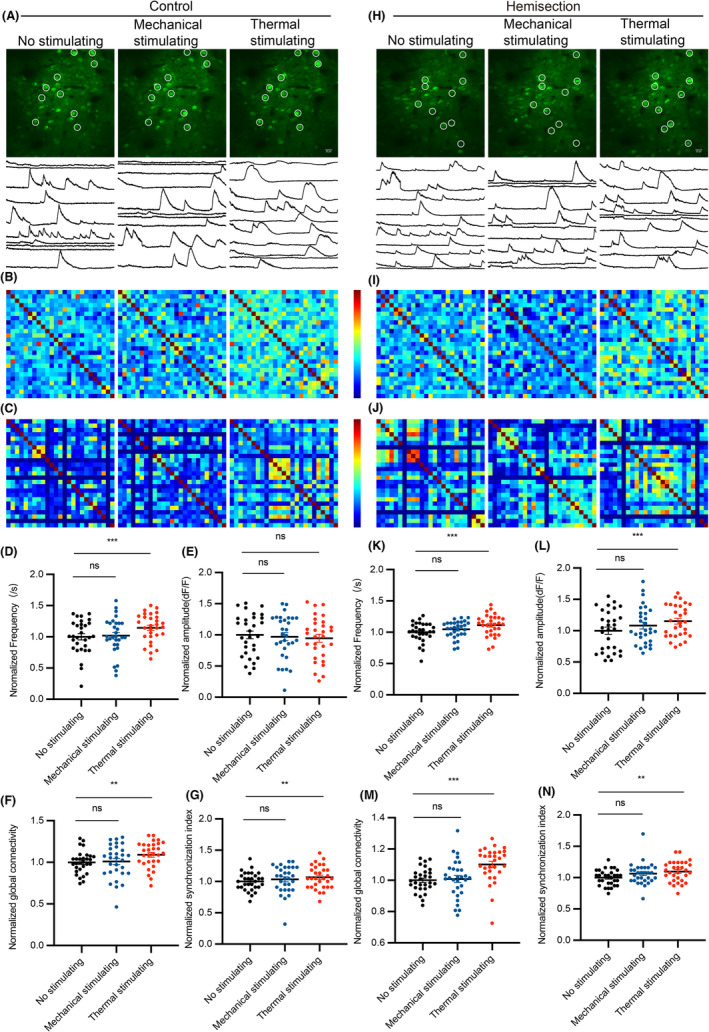
The right S1 neuronal activity increase was induced by thermal pain stimulation of the tail rather than mechanical pain stimulation of the right hind limb. (A, H) Example images of calcium transients as well as their neuronal calcium waves within right S1 in three phases before and after LHS. Circles represent typical neurons in individual microscope FOV. (B, I) Representative correlation matrix quantifying functional connectivity between each neuron and every other neuron within right S1 before and after LHS. (C, J) Representative correlation matrix quantifying network synchronization between each neuron and every other neuron within right S1 before and after LHS. (D–G) Frequency, amplitude, functional connectivity, and network synchronization of right S1 neuronal calcium transient waves in three phases before LHS (*n* = 750 neurons per 5 mice). (K–N) Frequency, amplitude, functional connectivity, and network synchronization of right S1 neuronal calcium transient waves in three phases after LHS (*n* = 750 neurons per 5 mice). All data were presented as mean ± s.e.m. and error bars represented s.e.m. All the data were tested for normal distribution followed by paired *t*‐test or nonparametric tests (Wilcoxon test). *p < 0.05, **p < 0.01, ***p < 0.001 and ****p < 0.0001.

**FIGURE 4:**
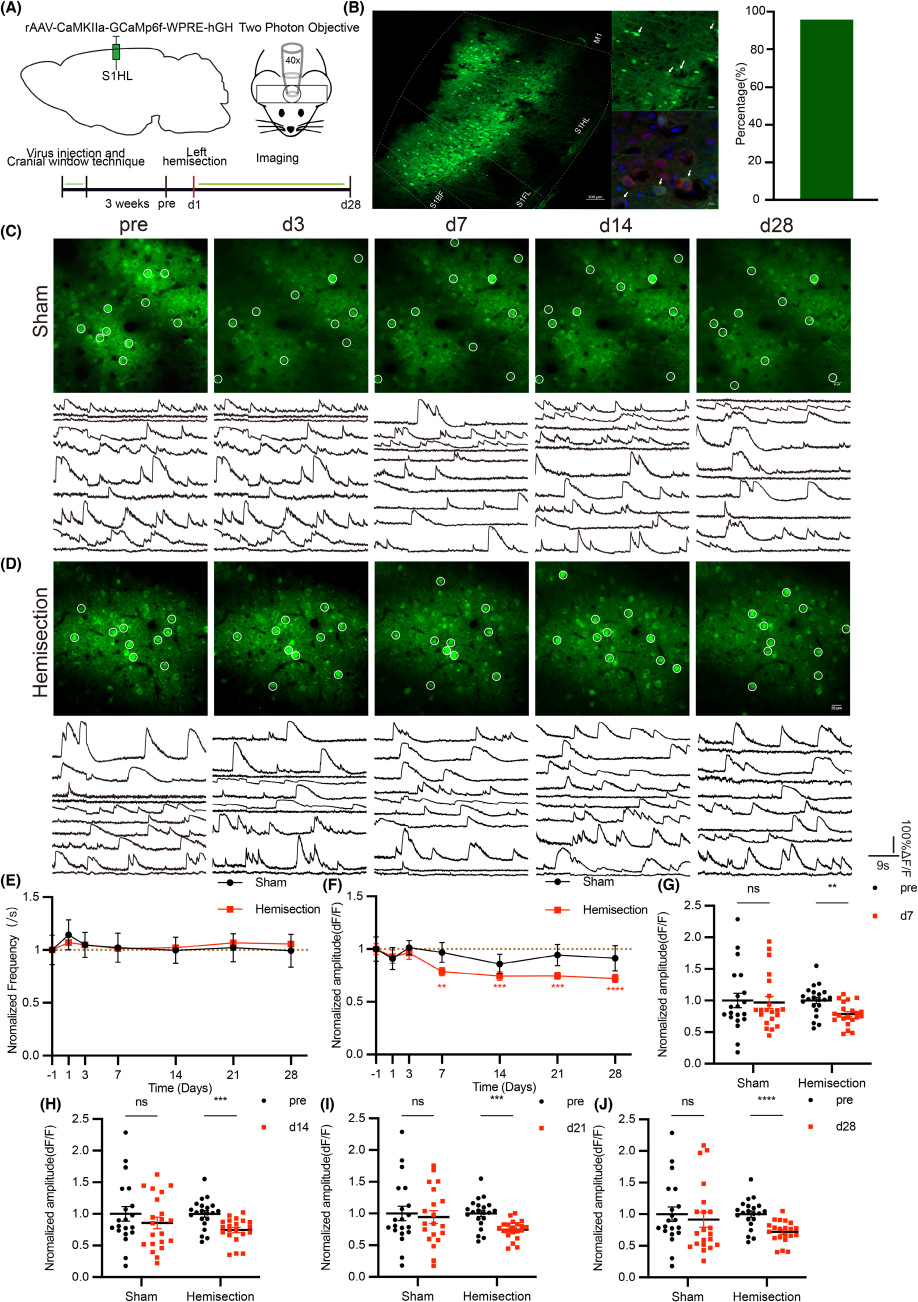
Frequency of calcium transients in left S1 has no change following LHS but amplitude is chronically declined. (A) Schematic diagram or timeline of virus injected region and in vivo imaging. (B) Left: Representative images of co‐labeling (arrowheads) of CaMKIIα‐positive neurons (red) and GCaMp6f (green) in the S1 (white dashed lines). Right: Proportion of co‐labeling neurons. (C, D) Example images of calcium transients within left S1 from sham and injured group as well as their neuronal calcium waves, traced at different time before and after LHS. Circles represent typical neurons in individual microscope FOV. (E, F) The statistics analysis of frequency and amplitude of left S1 neurons calcium transient waves in sham and injured group before and at specified time post‐LHS. (Sham group *n* = 500–525 neurons per 6 mice; Injured group *n* = 500–525 neurons per 7 mice). (G–J) Amplitude of left S1 neuronal calcium transient waves on day 7, 14, 21, and 28 in sham and injured group. (Sham group *n* = 500–525 neurons per 6 mice; Injured group *n* = 500–525 neurons per 7 mice). Data are mean ± s.e.m. and dots represent data points from individual microscope FOV from several animals. All the data were tested for normal distribution followed by unpaired *t*‐test or nonparametric tests (Mann–Whitney test). **p < 0.01. ***p < 0.001 and ****p < 0.0001.

To observe the alteration of calcium transients in S1 during mechanical and thermal stimulation, rAAV‐CaMKIIa‐GCaMP6f virus was injected into the layer 2/3 of S1 of hindlimb region (S1HL) and a chronic cranial window on the S1 was established 3 weeks in advance (Figure 4A). We measured left and right S1 excitatory neurons calcium transients during mechanical and thermal stimulation before and after LHS. Specifically, we recorded calcium transients for 45 s in the no‐stimulus state, followed by 45 s in the persistent mechanical stimulation (strength of filament to right hind limb:1.5 g). About 1 min later, calcium transients during thermal stimulation (tail in 52°C water) were recorded. Using vascular as landmarks, calcium transients from the same S1 neurons population were repeatedly observed and the data in the state of no stimulation were considered as baseline. (Figure 2A,H, Figure 3A,H). The calcium transients during stimulation were compared with baseline in control mice and at 7 days after LHS. We performed data analysis of the frequency, amplitude, functional connectivity (Figure 2B,I, Figure 3B,I), and network synchronization (Figure 2C,J, Figure 3C,J) of calcium transients. We found that in left S1, frequency, functional connectivity, and network synchronization were all increased except amplitude during mechanical stimulation of right hind limb and thermal stimulation of tail (Figure 2D–G). In right S1, these indicators increased only with thermal stimulation of tail but did not change with mechanical stimulation of right hind limb (Figure 3D–G). Moreover, the changes in S1 neuronal activity caused by pain stimulation were enhanced after spinal cord hemisection (Figure 2K–N, Figure 3K–N). These results suggest that mechanical pain stimulation of the right hind limb increased contralateral S1 neuronal activity while thermal pain stimulation of the tail increased bilateral S1 neuronal activity, and this effect was slightly enhanced after LHS.

### 3.3 Chronic alteration of functional connectivity happens in excitatory neurons of left S1 after spinal cord hemisection

**FIGURE 5:**
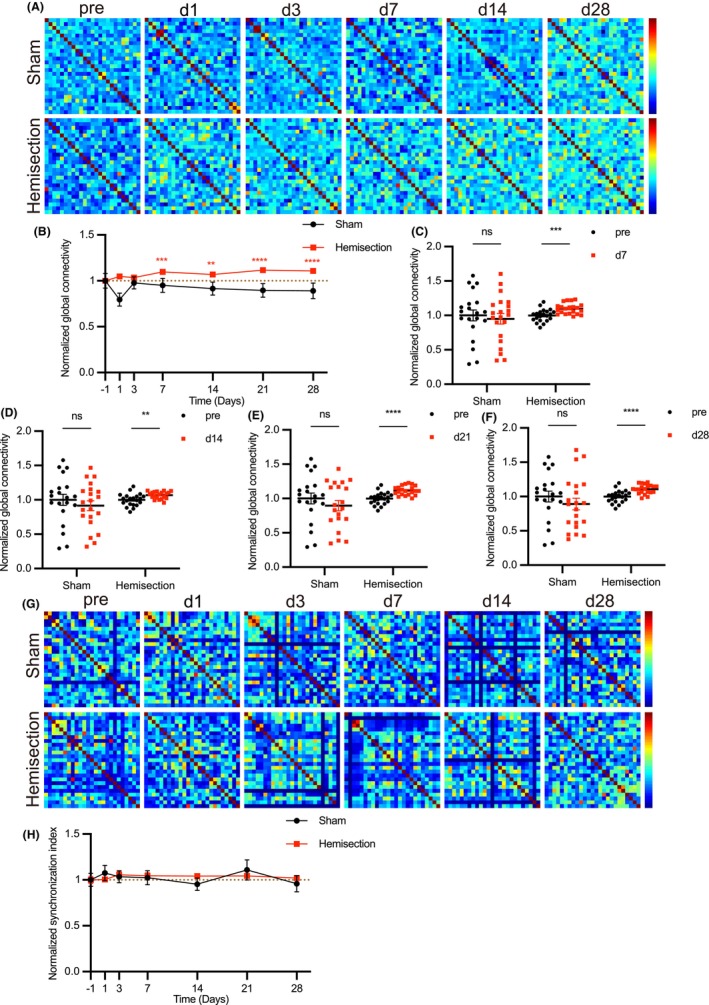
A significant increase in functional connection of excitatory neurons within left somatosensory cortex. (A) Representative correlation matrix quantifying functional connectivity between each neuron and every other neuron within left S1, traced at different time before and after LHS. (B) The statistics analysis of left S1 neurons functional connectivity in sham and injured group before and at specified time post‐LHS. (Sham group *n* = 500–525 neurons per 6 mice; Injured group *n* = 500–525 neurons per 7 mice). (C–F) Functional connectivity of calcium transients within left S1 in sham and injured group on day 7, 14, 21, and 28. (Sham group *n* = 500–525 neurons per 6 mice; Injured group *n* = 500–525 neurons per 7 mice). (G) Representative correlation matrix quantifying network synchronization between each neuron and every other neuron within left S1, traced at different time before and after LHS. (H) The statistics analysis of left S1 neurons activity synchronization index in sham and injured group before and at specified time post‐LHS. (Sham group *n* = 500–525 neurons per 6 mice; Injured group *n* = 500–525 neurons per 7 mice). Data are mean ± s.e.m. and dots represent data points from individual microscope FOV from several animals. All the data were tested for normal distribution followed by unpaired *t*‐test or nonparametric tests (Mann–Whitney test). **p < 0.01. ***p < 0.001 and ****p < 0.0001.

To observe the spontaneous alteration of calcium transients in S1 after LHS, we injected rAAV‐CaMKIIa‐GCaMP6f virus into the layer 2/3 of S1 of hindlimb region (S1HL) to detect characterized single‐cell calcium kinetics and network‐leveled features of calcium dynamics, including functional connectivity and network synchronization (Figure 4A). The virus was specifically expressed in excitatory neurons in the S1 (Figure 4B). Then a chronic cranial window on the S1 was established, which allowed us to observe calcium transients in awake mice for long periods of time (Figure 4A). After 3 weeks of waiting for virus expression, we performed two‐photon in vivo calcium imaging in mice before and after LHS (Figure 4A). Using vascular as landmarks, we were able to image spontaneous calcium transients repeatedly from the same S1 neurons population within 4 weeks (Figure 4C,D). Calcium imaging could simultaneously record the calcium transients of a neuron population in the visual field, which reflected the temporal activity of neurons in this area. We analyzed calcium transient waves that were characterized by a sharp rise time followed by a slower decay time (Figure 4C,D), including their frequency and amplitude. We took the data before surgery as the baseline value and then divided the data at each time point by it, which were normalized data. Then we compared these normalized data of each time point with those before operation. We found that the frequency of calcium transient in left S1 of injured mice had not changed during four weeks observation (Figure 4E and Figure S1A–F), whereas the amplitude was reduced on day 7 and it lasted to day 28 (Figure 4F–J and Figure S1G,H). Then, we analyzed functional connectivity and network synchronization of neuronal activity in this region (Figure 5A,G). We found that functional connectivity was enhanced after 7 days of LHS (Figure 5B–F and Figure S1I,J) but there was no change in neuronal firing synchronicity (Figure 5H and Figure S1K–P). Although the amplitude of calcium transient was chronically decreased, the functional connectivity in the left S1 excitatory neurons was gradually increased. The enhancement of functional connectivity is key characteristic of the alterations of pain microcircuit, which refers to a group of distributed neuronal assemblies bind to functional units so that they can transfer information more easily. In contrast, no obvious change happened in above indicators in sham group. These results suggest that only mild and chronic changes happen in left S1 after left spinal cord hemisection.

### 3.4 Significant and persistent alterations of calcium transients happen in excitatory neurons of right S1 after spinal cord hemisection

**FIGURE 6:**
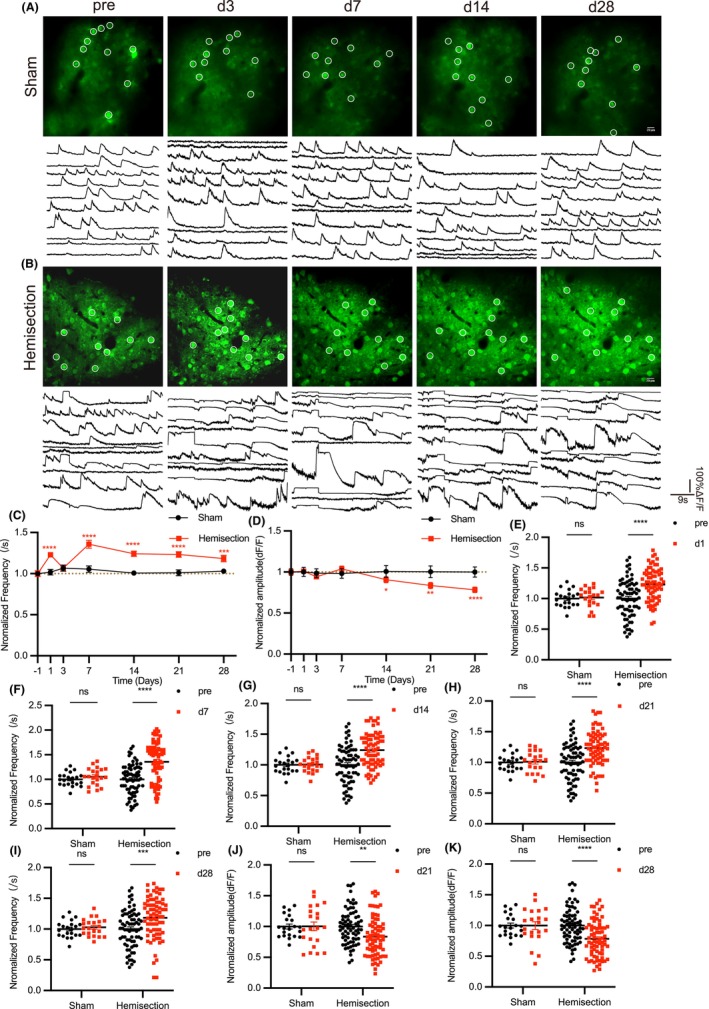
There are significant alterations in basal characteristics of the neuronal calcium transient in right somatosensory cortex. (A, B) Example images of calcium transient within right S1 from sham and injured group as well as their neuronal calcium waves, traced at different time before and after LHS. Circles represent typical neurons in individual microscope FOV. (C, D) The statistics analysis of frequency and amplitude of right S1 neuronal calcium transient waves in sham and injured group before and at specified time post‐LHS. (Sham group *n* = 500–525 neurons per 6 mice; Injured group *n* = 1675–1775 neurons per 9 mice). (E–I) Frequency of right S1 neuronal calcium transient waves on day 1, 7, 14, 21, and 28 in sham and injured group. (Sham group *n* = 500–525 neurons per 6 mice; Injured group *n* = 1675–1775 neurons per 9 mice). (J, K) Amplitude of right S1 neuronal calcium transient waves on day 21 and 28 in sham and injured group. (Sham group *n* = 500–525 neurons per 6 mice; Injured group *n* = 1675–1775 neurons per 9 mice). Data are mean ± s.e.m. and dots represent data points from individual microscope FOV from several animals. All the data were tested for normal distribution followed by unpaired *t*‐test or nonparametric tests (Mann–Whitney test). *p < 0.05. **p < 0.01. ***p < 0.001 and ****p < 0.0001.

**FIGURE 7:**
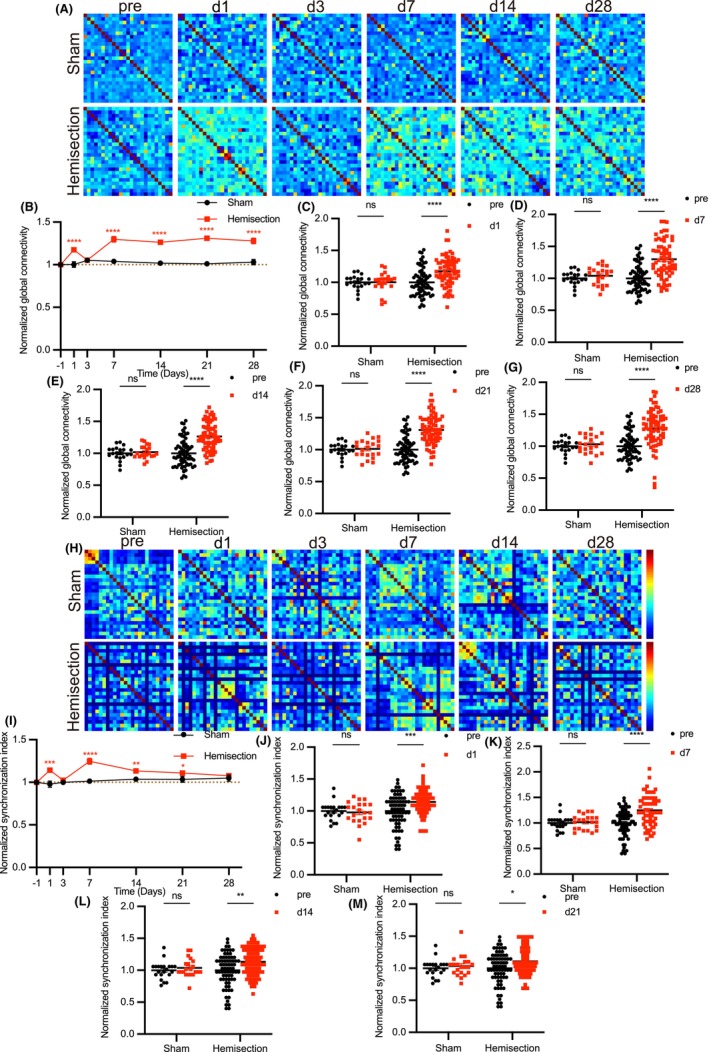
Increased functional connectivity and network synchronization of right S1 neurons in injured mice. (A) Representative correlation matrix quantifying functional connectivity between each neuron and every other neuron within right S1, traced at different time before and after LHS. (B) The statistics analysis of right S1 neurons functional connectivity in sham and injured group before and at specified time post‐LHS. (Sham group *n* = 500–525 neurons per 6 mice; Injured group *n* = 1675–1775 neurons per 9 mice). (C–G) Functional connectivity of calcium transients within right S1 in sham and injured group on day 1, 7, 14, 21, and 28. (Sham group *n* = 500–525 neurons per 6 mice; Injured group *n* = 1675–1775 neurons per 9 mice). (H) Representative correlation matrix quantifying network synchronization between each neuron and every other neuron within right S1, traced at different time before and after LHS. (I) The statistics analysis of right S1 neurons activity synchronization index in sham and injured group before and at specified time post‐LHS. (Sham group *n* = 500–525 neurons per 6 mice; Injured group *n* = 1675–1775 neurons per 9 mice). (J–M) Network synchronization within right S1 in sham and injured group on day 1, 7, 14, and 21. (Sham group *n* = 500–525 neurons per 6 mice; Injured group *n* = 1675–1775 neurons per 9 mice). Data are mean ± s.e.m. and dots represent data points from individual microscope FOV from several animals. All the data were tested for normal distribution followed by unpaired *t*‐test or nonparametric tests (Mann–Whitney test). *p < 0.05. **p < 0.01. ***p < 0.001 and ****p < 0.0001.

It is widely known that most spinal projection neurons target contralateral brain, including pathways that conduct pain in the skin and mucosa.^41^And damage of sensory ascending pathway in mammals is able to result in brain reorganization.^42^ Using the same experimental procedure and data processing method described above, we performed two‐photon in vivo calcium imaging of the right S1 (Figure 6A,B). We found that the frequency of calcium transient waves in injured mice was significantly elevated the day after injury. Then this effect returned to baseline level on day 3, but on day 7 it returned to high level and persisted for a long time (Figure 6C,E–I, and Figure S2A). Similar trend was observed in functional connectivity in injured group (Figure 7B–G and Figure S2F). Another important indicator is network synchronization, which is defined as a strong temporal correlation of calcium transients between two neurons in certain area (Figure 7H). It had a similar trend before d21, but returned to baseline on d28 (Figure 5I–M and Figure S2G,H). Moreover, the amplitude of calcium transient waves was declined 2 weeks after injury (Figure 6D,J,K and Figure S2B–E). In sham group, indicators of calcium activity were all around baseline and did not change significantly over four weeks. These data suggest that there are more significant alterations in the contralateral S1 after left spinal cord resection. Although the amplitude of calcium transient is also chronically decreased in right S1, the enhancements of frequency, function connectivity, and synchronization appear that there is some specific pattern of increased activity in right S1, in which groups of distributed neurons form into functional units in order to transfer information more efficiently.

### 3.5 Increased activity of excitatory lPBN neurons in neuropathic allodynia/hyperalgesia after spinal cord hemisection

**FIGURE 8:**
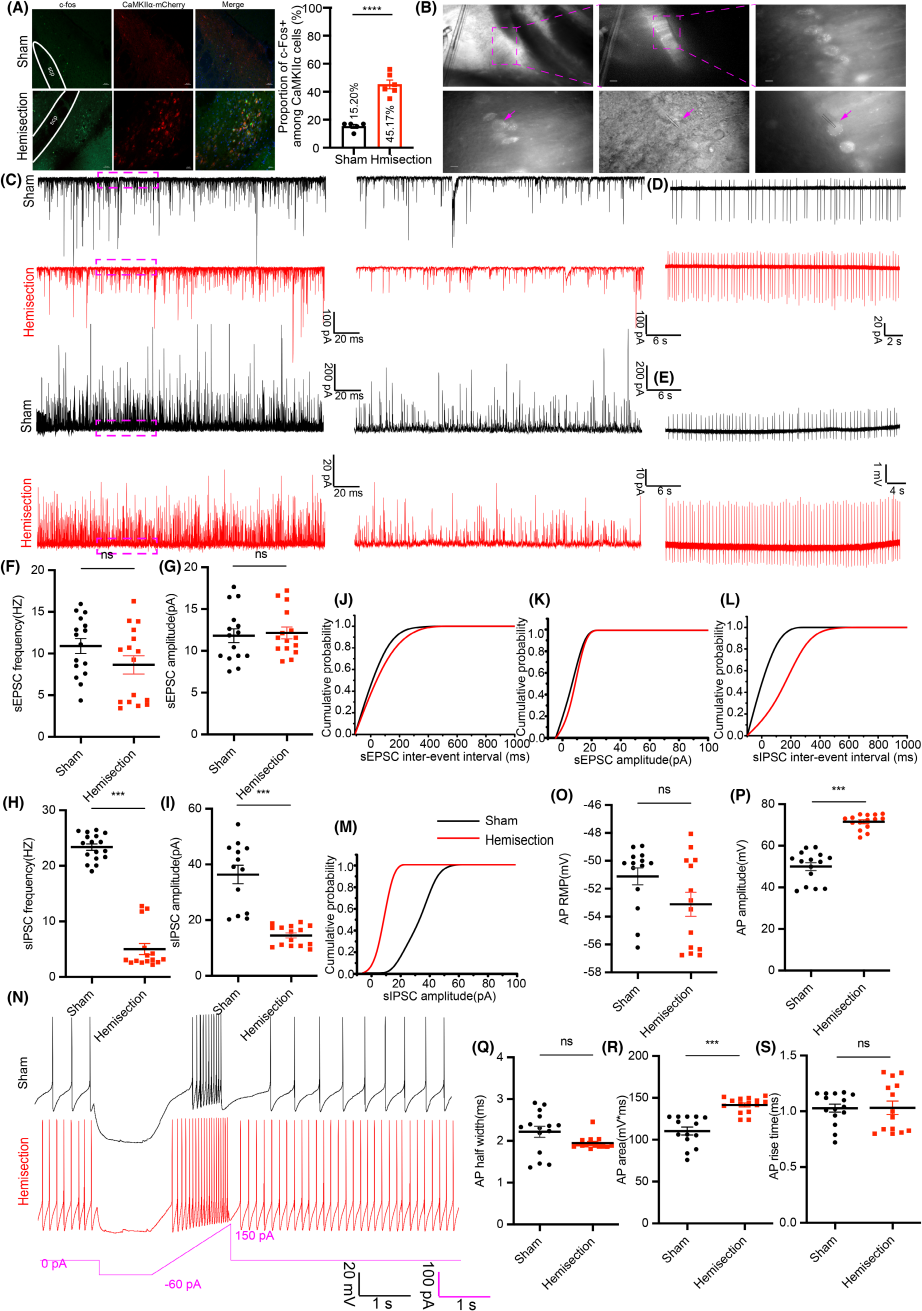
Increased excitability of CaMKIIα‐mCherry labeled neurons in lPBN after spinal cord hemisection. (A) Left: Representative immunofluorescence for c‐fos in sham and injured group. Right: Proportions of CaMKIIα positive cells co‐expressed with c‐Fos in the lPBN (Sham group 5 sections from 5 mice; Injured group 5 sections from 6 mice). (B) Top: representative CaMKIIα‐mCherry labeled neurons in lPBN. Bottom: representative electrophysiological recordings of glass microelectrodes on CaMKIIα‐mCherry labeled neurons. Arrows represented labeled neurons or microelectrode clamp cells. (C) Spontaneous EPSC (sEPSC) and sIPSC were recorded from sham (black traces) and injured (red traces) group. Right: Representative recording traces showing currents (purple dotted box). (D, E) Spontaneous intrinsic firing recorded with cell‐attached current clamp and cell‐attached voltage clamp in sham (black traces) and injured group (red traces). (F, G) Mean frequency and amplitude of sEPSCs were similar between sham and injured group (Sham group *n* = 4 sections from 4 mice; Injured group *n* = 5 sections from 4mice). (H, I) Mean frequency and amplitude of sIPSCs showed a significant decrease in injured group compared to sham group (Sham group *n* = 4 sections from 4 mice; Injured group *n* = 5 sections from 4 mice). (J, K) Cumulative distribution of sEPSC inter‐event interval and amplitude showed no significant difference between sham and injured group. (L, M) Cumulative distribution of sIPSC inter‐event interval and amplitude changed in injured group compared to sham group. (N) APs firing properties of lPBN CaMKIIα‐mCherry labeled neurons were altered after spinal cord hemisection. Representative traces of APs induced by injection of depolarizing current (ramp，0.14pA/ms) after hyperpolarizing the membrane (−60 pA for 1 s)，and then recording the spontaneous APs from sham (black traces) and injured (red traces) group. (O, Q, S) Resting membrane potential (RMP), AP half‐width, and AP rise time did not change between sham and injured group (Sham group *n* = 4 sections from 4 mice; Injured group *n* = 5 sections from 4 mice). (P, R) AP amplitude and AP area was increased in injured group compared to sham group (Sham group *n* = 4 sections from 4 mice; Injured group *n* = 5 sections from 4 mice). All data were presented as mean ± s.e.m. All the data were tested for normal distribution followed by unpaired *t*‐test or nonparametric tests (Mann–Whitney test). ***p < 0.001 and ****p < 0.0001.

Successful induction of mechanical allodynia of right hind limb and thermal hyperalgesia of tail was confirmed 7 days after LHS by behavior tests (Figure 1D–G). Immunostaining of c‐Fos 7 days after left spinal cord hemisection showed a dramatic increase in the density of c‐Fos positive cells co‐expressing CaMKIIα neurons in the lPBN as compared with sham group (Figure 8A). Previous research has shown that CaMKIIα^+^ and Vglut2^+^ neurons overlap extensively in the lPBN,^24^ so CaMKIIα^+^ neurons can be regarded as excitatory neurons. To dissect the physiological mechanisms underlying the overall increase in the expression of c‐fos during pain as described above, we used slice patch‐clamp recordings from injured and sham‐treated mice. Excitatory neurons were highly labeled in advance by local injection of adenovirus‐associated virus (pAAV‐CaMKIIα‐mCherry) into the lPBN (Figure 8B). 3 weeks after transduction, we used whole‐cell voltage clamp recordings to measure the spontaneous excitatory postsynaptic currents (sEPSC) and inhibitory postsynaptic currents (sIPSC) in excitatory neurons from sham or injured group (Figure 8C). We found that the frequency and amplitude of sIPSCs were significantly decreased in slices from spinal cord hemisection‐treated mice (Figure 8H,I), whereas there was no variation in frequency and amplitude of sEPSCs (Figure 8,G). Meanwhile, the cumulative amplitude distribution of sIPSC shifted to the left, and interevent interval shifted to the right (Figure 8L,M), while the inter‐event interval and amplitude of sEPSC did not shift significantly between the two groups. (Figure 8J,K). The decrease in the frequency and amplitude of spontaneous inhibitory events suggests that the excitability of lPBN excitatory neurons were significantly enhanced in pain hypersensitivity after LHS. To test whether central nerve pain modulates lPBN neuron firing, we performed whole‐cell current clamp recordings in lPBN excitatory neurons in injured treated and sham mice (Figure 8N). We found that the action potential (AP) amplitude and area were significantly increased after LHS (Figure 8P,R). However, there was no difference in the AP reseing membrane potential (RMP), the half‐width and the rise time as compared with sham group (Figure 8O,Q,S). In order to record the excitability of neurons closer to the physiological state, cell‐attached recording was used to detect the neuronal firing in lPBN. We found that the spontaneous firing of excitatory neurons was more frequent after LHS in both current‐clamp and voltage‐clamp modes. (Figure 8D,E). These results suggest that the excitability of lPBN excitatory neurons was significantly increased in the state of allodynia and hyperalgesia.

### 3.6 Activating or inhibiting lPBN excitatory neurons can simulate or alleviate allodynia and hyperalgesia after spinal cord hemisection

**FIGURE 9:**
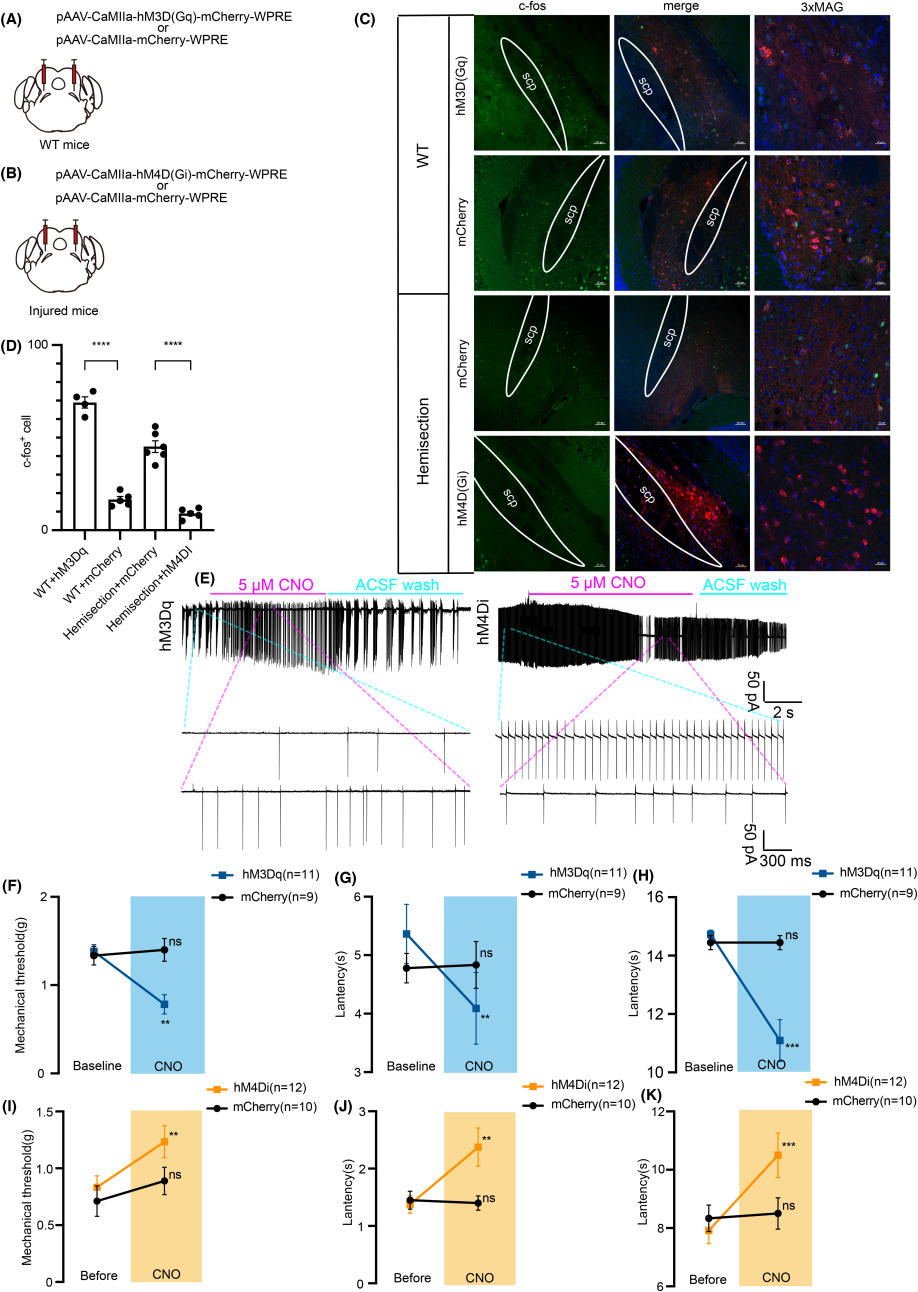
Allodynia and hyperalgesia following LHS can be simulated or mitigated by modulating lPBN excitatory neurons. (A) Schematic of the virus injection strategy for the expression of CaMKIIα‐hm3Dq or CaMKIIα‐mCherry in the lPBN of wild‐type mice. (B) Schematic of the virus injection strategy for the expression of CaMKIIα‐hm4Di or CaMKIIα‐mCherry in the lPBN of injured mice. (C) Representative immunofluorescence for c‐fos in WT and injured mice. (D) CNO‐mediated activation of hM3Dq could enhance expression of c‐fos in lPBN in WT mice and activation of hM4Di could deplete expression of c‐fos in lPBN in injured mice (WT + hm3Dq *n* = 4 sections from 4 mice; WT + mCherry *n* = 5 sections from 4mice; Hemisection+mCherry *n* = 6 sections from 5 mice; Hemisection+hM4Di *n* = 5 sections from 5 mice, unpaired *t*‐test). (E) Left: Spontaneous intrinsic firing increased after application of 5 μM CNO in the CaMKIIα‐hM3Dq labeled PBN neurons. Right: Spontaneous intrinsic firing decreased after application of 5 μM CNO in the CaMKIIα‐hM4Di labeled PBN neurons. Insets of light blue dotted line, representative trace is the spontaneous intrinsic firing recorded by cell‐attached voltage clamp before CNO administration. Insets of purple‐blue dotted line, representative trace is the spontaneous intrinsic firing recorded by cell‐attached voltage clamp after CNO administration. (F–H) CNO‐mediated activation of hM3Dq decreased the threshold of the paw‐withdrawal and latency of the tail to thermal stimulation in WT mice (hM3Dq *n* = 11 mice; mCherry *n* = 9 mice, paired *t*‐test). (I–K) CNO‐mediated activation of hM4Di increased the threshold of the paw withdrawal and latency of the tail to thermal stimulation in injured mice (hM4Di *n* = 12 mice; mCherry *n* = 10 mice, paired *t*‐test). All data were presented as mean ± s.e.m. All the data were tested for normal distribution followed by unpaired *t*‐test or paired *t*‐tests. **p < 0.01, ***p < 0.001 and ****p < 0.0001.

The above results that lPBN excitatory neurons were activated in the presence of allodynia and hyperalgesia following spinal cord hemisection. In contrast to many other ascending pathways, the spinal cord sends projections to bilateral PBN.^27^, ^31^, ^43^ To further examine the role of lPBN excitatory neurons in mediating neuropathic pain, we manipulated their activity using chemogenetic approach in wild mice (WT mice) and injured mice (Figure 9A,B). We bilaterally injected the lPBN of WT mice with an pAAV expressing hM3Dq (a designer receptor exclusively activated by designer drugs) and mCherry as control, which mimicked the activation of lPBN in natural mice (Figure 9A). Three weeks after virus infection, chemogenetic activation lPBN excitatory neurons via hM3Dq using clozapine N‐oxide (CNO) produced significant c‐fos increase in WT mice (Figure 9C,D). We then found that activation of excitatory lPBN neurons drastically decreased the threshold of the paw‐withdrawal responses evoked by mechanical stimulation (Figure 9F) and latency of the tail to noxious thermal stimulation (Figure 9G,H) in wild mice, while neurons transfected with mCherry had no effect (Figure 9F–H). Conversely, inhibiting lPBN excitatory neurons with hM4Di produced significant decrease in the expression of c‐fos in injured mice (Figure 9C,D). As expected, the decreased mechanical threshold and latency induced by LHS were elevated after the CNO injections in injured mice expressing hM4Di in the excitatory lPBN neurons, but not in mice transfected with mCherry (Figure 9I–K). CNO effects were confirmed again by slice electrophysiological recordings with cell‐attached. The application of hM3Dq agonist CNO (5 μM) increased the firing frequency and amplitude of lPBN excitatory neurons, when CNO was washed away by ACSF, the firing frequency decreased (Figure 9E left). In contrast, when CNO (5 μM) was perfused in brain slices from injured mice expressing hM4Di, the firing frequency and amplitude of excitatory neurons were decreased (Figure 9E right). These results demonstrate that excitatory lPBN neurons play an important role in maintenance of neuropathic pain after LHS. Performing activation or inhibition of lPBN excitatory neurons can induce or alleviate allodynia and hyperalgesia after spinal cord hemisection.

## 4 DISCUSSION

The transmission of pain information is mediated by the joint conduction of peripheral and spinal pathways,^44^, ^45^ but reorganizing and information processing of some central brain regions also play a very important role in the maintenance of abnormal pain.^46^, ^47^ The cortical regions have been recognized as a “pain matrix,” where neurons are activated during nociceptive stimuli.^48^, ^49^ And the lateral parabrachial nucleus is an important region closely related to neuropathic pain behavior.^27^, ^28^ Their role in development of central neuropathic pain was the focus of our research.

By mechanical and thermal stimulation‐related behavioral tests, we identified allodynia of right hind limb and hyperalgesia of tail following LHS. The mechanisms of mechanical allodynia/hyperalgesia and thermal allodynia/hyperalgesia are different. The Punctate allodynia/hyperalgesia (the type of mechanical allodynia/hyperalgesia in this study) is driven by activity in Aδ fibers and a minor input from C fibers,^50^ which depends on central changes in addition to peripheral input.^51^ Heat stimuli are conducted via C fibers and Aδ fibers and heat hyperalgesia is probably likewise a result of central mechanisms and is present in 10% of patients with central pain.^52^ In this chronic negative state, we investigated the temporal profile of spontaneous calcium transient on single neuron basis in S1 and electrophysiological characteristics in lPBN. In left S1, the frequency and network synchronization remained normal level though the amplitude of calcium transient was declined 7 days after injury, and interestingly, the functional connectivity was gradually enhanced. Whereas in right S1, except that the amplitude dropped to normal level after 3 weeks, the frequency, functional connectivity, and network synchronization of calcium transient were all significantly enhanced in long‐term detection after LHS (except for the day 3). Moreover, the calcium activity in left S1 was increased during mechanical stimulation of right hind limb and thermal stimulation of tail, whereas in right S1 it was increased only with heat stimulation of tail. In whole‐cell recording in lPBN excitatory neurons after LHS, the frequency and amplitude of sIPSCs were significantly decreased, and the AP amplitude and area were significantly increased. In cell‐attached recording, the spontaneous firing of excitatory neurons was more frequent. These results of whole‐cell recording mean that the inhibition input of lPBN excitatory neurons is reduced and these neurons are easy to exciting.

Neurons in network display intricate spontaneous and stimulus‐evoked spatiotemporal activity pattern in normal state. As calcium modulates excitability, neurotransmission, and other essential cellular processes,^53^ the temporal imaging of calcium transient within neurons provides an opportunity to have an insight into network structure. Reorganization of large‐scale resting‐state brain networks in traumatic SCI has been shown in human by functional MRI.^54^ Imaging calcium in neurons has become popular for investigating activity of neuron populations and has revealed neuronal connection maps within a certain field of view.^55^, ^56^ In neuroanatomy, the Latin word “homunculus” represents the sensory distribution along the cerebral cortex of the brain,^57^ which means that the projection of superficial sensations has a precise correspondence in the cerebral cortex. Previous research showed that cortical synaptic rewiring could be sufficient to cause potential mirror image pain after peripheral nerve injury,^22^ but they did not elucidate the role of increased functional connection within S1 in the production of ectopic pain. Therefore, in this study, the increased functional connection in left S1 is likely to induce mechanical allodynia of right hind limb. Calcium transient reflects the spontaneous electrical activity of neurons, which is initiated by synaptic activity and the associated opening of gated ion channels.^58^ These activities induce structural remodeling in neurites, synapses, dendritic spines, which is the basis of increased functional connection.^59^ Due to the paralysis of the left hind limb, we cannot measure their response to nocuous stimulus, but the thermal hyperalgesia of tail is likely related to the abnormal activity in the bilateral S1 neuronal network. Increased calcium signal reflects neural dysfunction and relate to the reorganization of S1, to some extent, relate to aberrant feeling such as pathological pain, hyperalgesia, and phantom limb pain.^60^ Although the mechanism of cortical reorganization following SCI remains unclear so far, studies have shown that the sprouting of intact neurons into the deafferented cortex is associated with cortical reorganization.^61^, ^62^ Deafferentation caused by SCI immediately change the state of the cortical network, resulting in an increased cortical response to stimuli above the level of injury.^63^ Changes over a longer time likely involve other additional mechanisms such as long‐term potentiation, axonal regeneration and sprouting.^61^ These trends are also reflected in this study. Moreover, SCI has variable effects on cortical activities, range from mild alteration of sensory activity in incomplete injury to massive reorganization of sensory network in complete injury, suggesting cortical reorganization is highly variable.^64^

However, the cortico‐centric view of neuropathic pain may overlook the fundamental idea that avoiding tissue damage is a primal need in which subcortical pathways play a central role.^28^ In this study, we found that the c‐fos expression was increased in lPBN 7 days after LHS, which indicated that the whole region was in activated state. By slice patch clamp recording, the electrophysiological characteristics of individual excitatory neurons were clarified. The decreased frequency and amplitude of IPSC, with increased amplitude and area of evoked action potential, further verified its increased activity at the cellular level. These effects of this injury are bilateral, which is in line with the previous conclusion that spinal cord sends projections to bilateral PBN.^27^, ^31^ Considering the important role of lPBN in pain management and regulation, we proposed a hypothesis that regulating lPBN excitatory neurons could change the present of allodynia and hyperalgesia. As expected, using bilaterally chemogenetic activation of lPBN excitatory neurons was sufficient to induce allodynia/hyperalgesia in wild mice, whereas inhibiting their activity could relieve this state in injured mice. CNO‐mediated activation of hM3Dq could enhance neuronal firing in lPBN excitatory neurons, which simulated the active state after a spinal cord injury. While activation of hM4Di silencing lPBN excitatory neurons could alleviate allodynia/hyperalgesia in injured mice. Previous studies have shown that the ratio of excitatory neurons in lPBN is nearly 90 percent,^24^ and the majority respond to noxious stimuli.^25^, ^65^, ^66^ The lPBN is a primary target for nociceptive information arising from the spinal cord,^67^, ^68^, ^69^, ^70^ and this region conveys nociceptive input into supraspinal structures.^28^, ^71^ In this study, lPBN plays critical role in central neuropathic pain development.

In summary, we demonstrated the differentiated cortical reorganizations and the important role of lPBN in development of central neuropathic pain. More studies are needed to further examine important circuits in supraspinal structures, which is underlying mechanism leading to reorganizations of the cortex. All these will provide a theoretical basis for treating aberrant feeling and help develop new therapeutic strategies for SCI.

## FUNDING INFORMATION

This work was supported by the National Natural Science Foundation of China (Grant Nos. 62027812), the Joint Funds for the National Key Research and Development Program of China (Grant No.2021ZD0202900), the National Natural Science Foundation of China (81771470, 81871776 and 82101608).

## CONFLICT OF INTEREST STATEMENT

The author (s) declared no conflicts of interest regarding this article.

## Supporting information


Figure S1–S2:

Video S1:

## Data Availability

The data that support the findings of this study are available from the corresponding author upon reasonable request.
