# Supplementary material for: Neuropathic pain following spinal cord hemisection induced by the reorganization in primary somatosensory cortex and regulated by neuronal activity of lateral parabrachial nucleus
Source: CNS Neurosci Ther. 2023 May 11;29(11):3269–89. doi: 10.1111/cns.14258 (PMC10580357; doi:10.1111/cns.14258)
Supplement: Supplementary file 1 — Figure S1–S2: [file CNS-29-3269-s002.docx]

Supplementary Figure 1. Frequency and network synchronization of calcium transients have no obvious change in left S1 after LHS.

(A-F) Frequency of left S1 neuronal calcium transient waves on day 1, 3, 7, 14, 21 and 28 in sham and injured group. (Sham group n = 500-525 neurons per 6 mice; Injured group n= 500-525 neurons per 7 mice). (G, H) Amplitude of left S1 neuronal calcium transient waves on day 1and 3 in sham and injured group. (Sham group n = 500-525 neurons per 6 mice; Injured group n= 500-525 neurons per 7 mice). (I, J) Functional connectivity of calcium transients within left S1 in sham and injured group on day 1 and 3. (Sham group n = 500-525 neurons per 6 mice; Injured group n= 500-525 neurons per 7 mice). (K-P) Network synchronization of calcium transients within left S1 in sham and injured group on day 1, 3, 7, 14, 21 and 28. (Sham group n = 500-525 neurons per 6 mice; Injured group n= 500-525 neurons per 7 mice). Data are mean ± s.e.m. and dots represent data points from individual microscope FOV from several animals. All the data were tested for normal distribution followed by un-paired t test or nonparametric tests (Mann-Whitney test). **P* < 0.05. ***P* < 0.01. ****P* < 0.001 and *****P* < 0.0001.


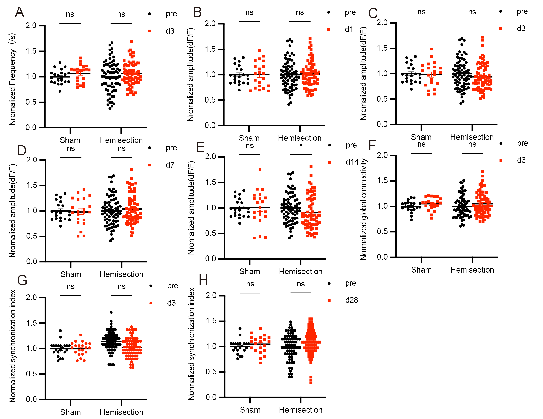


Supplementary Figure 2. Frequency, functional connectivity and network synchronization calcium transients return to baseline level on day 3 in right S1 after LHS.

(A) Frequency of right S1 neuronal calcium transient waves on day 3 in sham and injured group. (Sham group n = 500-525 neurons per 6 mice; Injured group n= 1675-1775 neurons per 9 mice). (B-E) Amplitude of right S1 neuronal calcium transient waves on day1, 3, 7 and 14 in sham and injured group. (Sham group n = 500-525 neurons per 6 mice; Injured group n= 1675-1775 neurons per 9 mice). (F) Functional connectivity of calcium transients within right S1 in sham and injured group on day 3. (Sham group n = 500-525 neurons per 6 mice; Injured group n= 1675-1775 neurons per 9 mice). (G, H) Network synchronization within right S1 in sham and injured group on day 3 and 28. (Sham group n = 500-525 neurons per 6 mice; Injured group n= 1675-1775 neurons per 9 mice. Data are mean ± s.e.m. and dots represent data points from individual microscope FOV from several animals. All the data were tested for normal distribution followed by un-paired t test or nonparametric tests (Mann-Whitney test). **P* < 0.05. ***P* < 0.01. ****P* < 0.001 and *****P* < 0.0001.
